# Welcome to volume 6 of *Future Science OA*

**DOI:** 10.2144/fsoa-2019-0148

**Published:** 2019-12-16

**Authors:** Francesca Lake

**Affiliations:** 1 Future Science Group, Unitec House, 2 Albert Place, London, N3 1QB, UK

Welcome to the first issue of volume 6 from *Future Science OA*! In this Foreword, I will take a look over both the highlights of 2019 in *Future Science OA* and what we can expect from 2020.

The year of 2019 was a fantastic year for the journal, with it becoming indexed on Scopus and seeing an average of 38,000 full-text readers a month across our approximately 450 publications.

Those readers come to access great research and we published some really interesting articles in 2019 from a wide range of topics. My personal highlight was an article entitled “‘Academic periodization’: using approaches from elite sport to benefit early career academics” by J Gonzalez and K Deighton [1]. This article was part of a special issue focused on early career researchers and discussed how periodization – a method used by athletes to maximize performance while minimizing risk of overtraining and injury – can be applied to early career researchers. This article formed part of an excellent issue guest edited by L Heaney (Loughborough University, UK), one of our panel of Young Ambassadors, and is well worth a read for any researcher looking to advance their career without burning out [2].

Another fantastic article was a review entitled “Bromodomain and extra-terminal motif inhibitors: a review of preclinical and clinical advances in cancer therapy” by Alqahtani *et al* [3]. This is our second most-read article from 2019 (after [1]) and makes for a fascinating read.

These articles are by no means my only highlights from this year – it is hard to pick from the over 70 new research, review and opinion pieces we have published this year, as well as the novel methodologies and data notes!

We also supported the third iteration of the Future Science Early Career Research Award, which has been renamed the Future Science Future Star Award. This year, M Pizarro-Guajardo (Universidad Andrés Bello, Santiago, Chile) won, following stiff competition from 28 candidates [4]. She will be guest editing a special issue of *Future Science OA*, which will be published in 2020. In the meantime, you can find out more about her fantastic career so far in our winner’s podcast [5].

This year also saw the publication of a research article from last year’s winner, V Mucci [6]. Mucci’s study examined the physiological changes that occur during pregnancy for patients with Mal de Debarquement syndrome. We are delighted with how winning the award has increased visibility of research into this rare neurological disorder.

## Journal statistics

It currently takes, on average, 9 weeks from submission to acceptance of an article for publication in *Future Science OA*. The journal currently accepts 80.5% of submissions for publication. At the time of writing (all data were collected on 25 November 2019), the journal has received 787 citations in 2019 – an appreciable increase on 2018 (data taken from Dimensions [7]). This year, *Future Science OA* articles have also been mentioned in the news 44-times and the editorial team is always delighted to see articles being picked up and communicated to the public.

**Figure 1.**
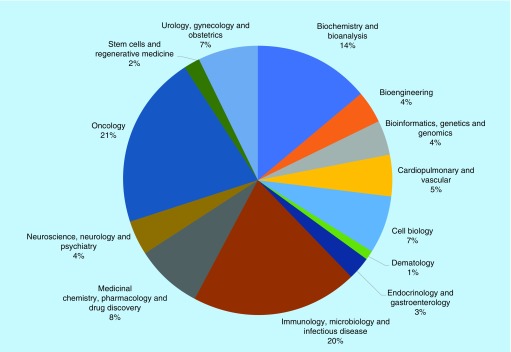
Topics covered in *Future Science OA* by percentage in 2019.

**Figure 2.**
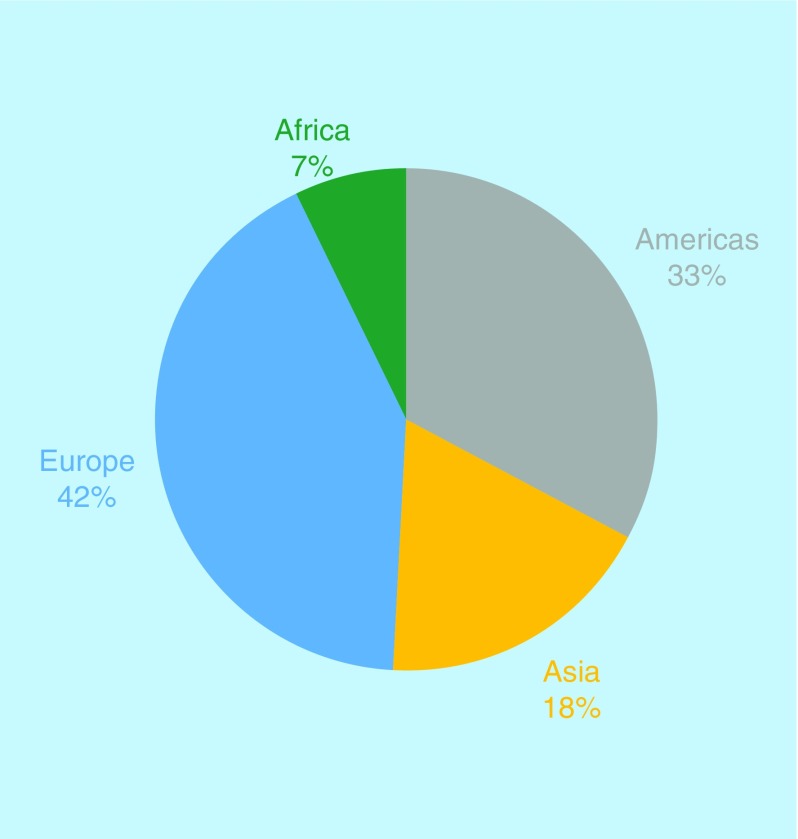
*Future Science OA* author demographics in 2019.

In terms of topic areas, this year the journal has continued last year’s trend [8], with a higher percentage of publications in the oncology and immunology/microbiology topic areas (Figure 1). This mirrors the state of the biomedical field, with oncology, immunology and infectious diseases remaining highly researched topic areas. Author demographics also remained fairly consistent, seeing a small decrease in the proportion of authors from the USA, in favor of Africa and Asia, which is something that has been made feasible by our fee waiver program (Figure 2).

**Figure 3.**
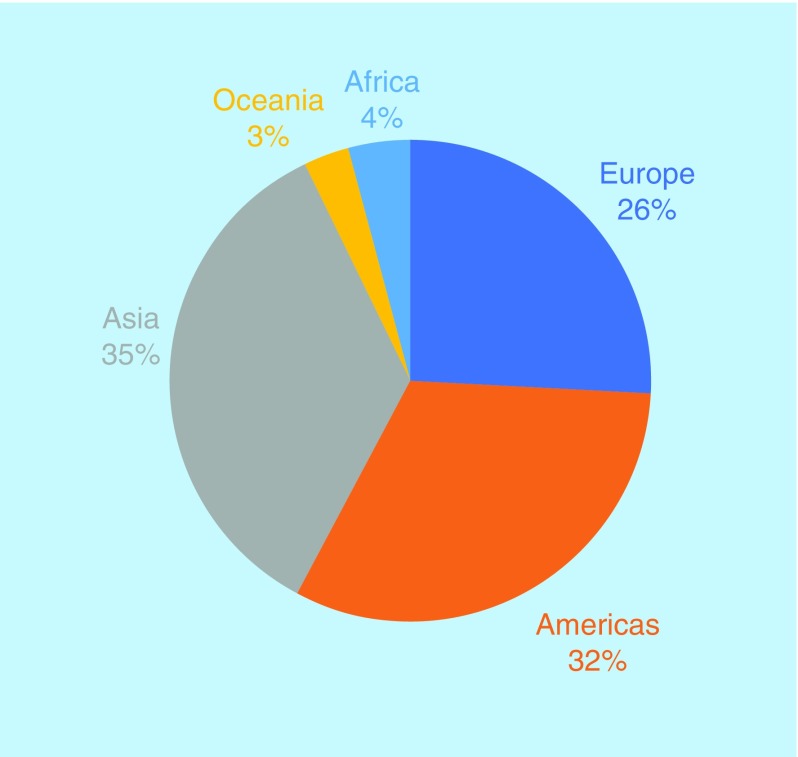
*Future Science OA* reader demographics in 2019.

The proportion of our readers from each continent has also remained relatively similar, with a small decrease in those from the USA and increase in those in Asia (Figure 3). It should be noted, however, that the number of readers is much higher year-on-year.

One final fact I would like to note is that since our launch in 2015, we have had 433 of our articles listed on ScienceOpen [9], which also tells us that those articles have referenced, and thus built upon 15,344 other articles. With reference lists so often hidden behind a paywall, it is fascinating to be able to see such contextual information for our articles.

## Thanks to our contributors

*Future Science OA* would not be able to succeed without the time investment made by our contributors – this includes our excellent editorial board as well as the thousands of authors and peer reviewers we have worked with since our launch in 2015.

## Looking forward to 2020

The year of 2019 has been fabulous and we have some excellent plans for 2020, too. We have recently begun hosting all of our supplementary information on Figshare, meaning that information is both easily available and citable, helping us to support the open data movement. We are also intending to integrate with bioRxiv, allowing those who post their preprints to submit straight to the journal, decreasing the time spent inputting information into submission systems. We will also be supporting the next iteration of the Future Science Future Star Award and publishing the thematic issue guest edited by this year’s winner. I look forward to working with you all!
